# Hospital based cross sectional study of herpes zoster with reference to HIV seropositivity

**DOI:** 10.1186/1471-2334-12-S1-P58

**Published:** 2012-05-04

**Authors:** Murugan Sundaram, S Adikrishnan, M Krishnakanth, R Sudha, V Mahalakshmi, S Shobana, S Anandan

**Affiliations:** 1 Dept. of Dermatology & S.T.D., Sri Ramachandra University, Chennai, India

## Background

Herpes zoster is a common viral infection caused by the reactivation of the latent Varicella zoster virus. There is an increase in the unusual clinical presentation of herpes zoster because of increasing trends of HIV infection. This work is done to study herpes zoster with regard to incidence, extent of involvement and assess HIV seropositivity.

## Methods

A one year cross sectional assessment of the patients with herpes zoster with reference to their age, sex, prodromal symptoms and distribution of lesions. A detailed history of blood transfusion, drug addiction, exposure to risk of sexually transmitted infections, other associated diseases like tuberculosis etc. were recorded. Screening for HIV was done in all patients.

## Results

The incidence of herpes zoster was 0.46% in the dermatology OP. The youngest patient was 4 years old and the oldest was 80 years. The incidence in males was 69.2% and females was 30.76%, Dermatomal distribution – thoracic dermatomes 55%, cranial dermatomes 21.5%, lumbar 18.4%, cervical 4.6% and sacral 1.5%. Overall, 32.3% of herpes zoster patients were HIV seropositive, the youngest seropositive patient was 8 years and the oldest being 56 years.

## Conclusion

Zoster may be the first sign of immune suppression due to HIV infection. Any patient presenting with severe multidermatomal herpes zoster with 5^th^ cranial nerve involvement and cervical segment involvement should be screened for underlying HIV infection. Hence screening for HIV in all case of herpes zoster is mandatory.

